# Spexin and Galanin in Metabolic Functions and Social Behaviors With a Focus on Non-Mammalian Vertebrates

**DOI:** 10.3389/fendo.2022.882772

**Published:** 2022-05-25

**Authors:** Izzati Mohd Zahir, Satoshi Ogawa, Nisha Angela Dominic, Tomoko Soga, Ishwar S. Parhar

**Affiliations:** ^1^ Brain Research Institute Monash Sunway, School of Medicine and Health Sciences, Monash University Malaysia, Subang Jaya, Malaysia; ^2^ Clinical School Johor Bahru, Monash University Malaysia, Johor Bahru, Malaysia

**Keywords:** neuropeptide, energy regulation, reproduction, stress, anxiety, depression, hypothalamus

## Abstract

Spexin (SPX) and galanin (GAL) are two neuropeptides that are phylogenetically related and have descended from a common ancestral gene. Considerable attention has been given to these two multifunctional neuropeptides because they share GAL receptors 1,2, and 3. Since GAL and SPX-synthesizing neurons have been detected in several brain areas, therefore, it can be speculated that SPX and GAL are involved in various neurophysiological functions. Several studies have shown the functions of these two neuropeptides in energy regulation, reproduction, and response to stress. SPX acts as a satiety factor to suppress food intake, while GAL has the opposite effect as an orexigenic factor. There is evidence that SPX acts as an inhibitor of reproductive functions by suppressing gonadotropin release, while GAL modulates the activity of gonadotropin-releasing hormone (GnRH) neurons in the brain and gonadotropic cells in the pituitary. SPX and GAL are responsive to stress. Furthermore, SPX can act as an anxiolytic factor, while GAL exerts anti-depressant and pro-depressive effects depending on the receptor it binds. This review describes evidence supporting the central roles of SPX and GAL neuropeptides in energy balance, reproduction, stress, and social behaviors, with a particular focus on non-mammalian vertebrate systems.

## 1 Introduction

Neuropeptides are essential signaling molecules that work to integrate information in the nervous systems. In the central nervous system, neuropeptides are especially important in the hypothalamic circuitry, as these molecules underlie both neural and non-neural communications as part of the neuroendocrine system. Two neuropeptides, namely, spexin (SPX) and galanin (GAL), have received considerable attention in recent years. Although GAL has been known for quite a while, SPX is newly discovered, and researchers have uncovered its potential functions. GAL has been implicated in various functions in the neuroendocrine system. Similarly, studies on SPX have identified its functions related to hypothalamic circuitry. It was found that SPX can bind to some GAL receptors, thus, linking these two neuropeptides in addition to their common ancestral origin (1). As such, GAL and SPX are involved in similar processes, namely, in energy regulation, reproduction, stress responses and mood disorders (2, 3).

**Figure 1:**
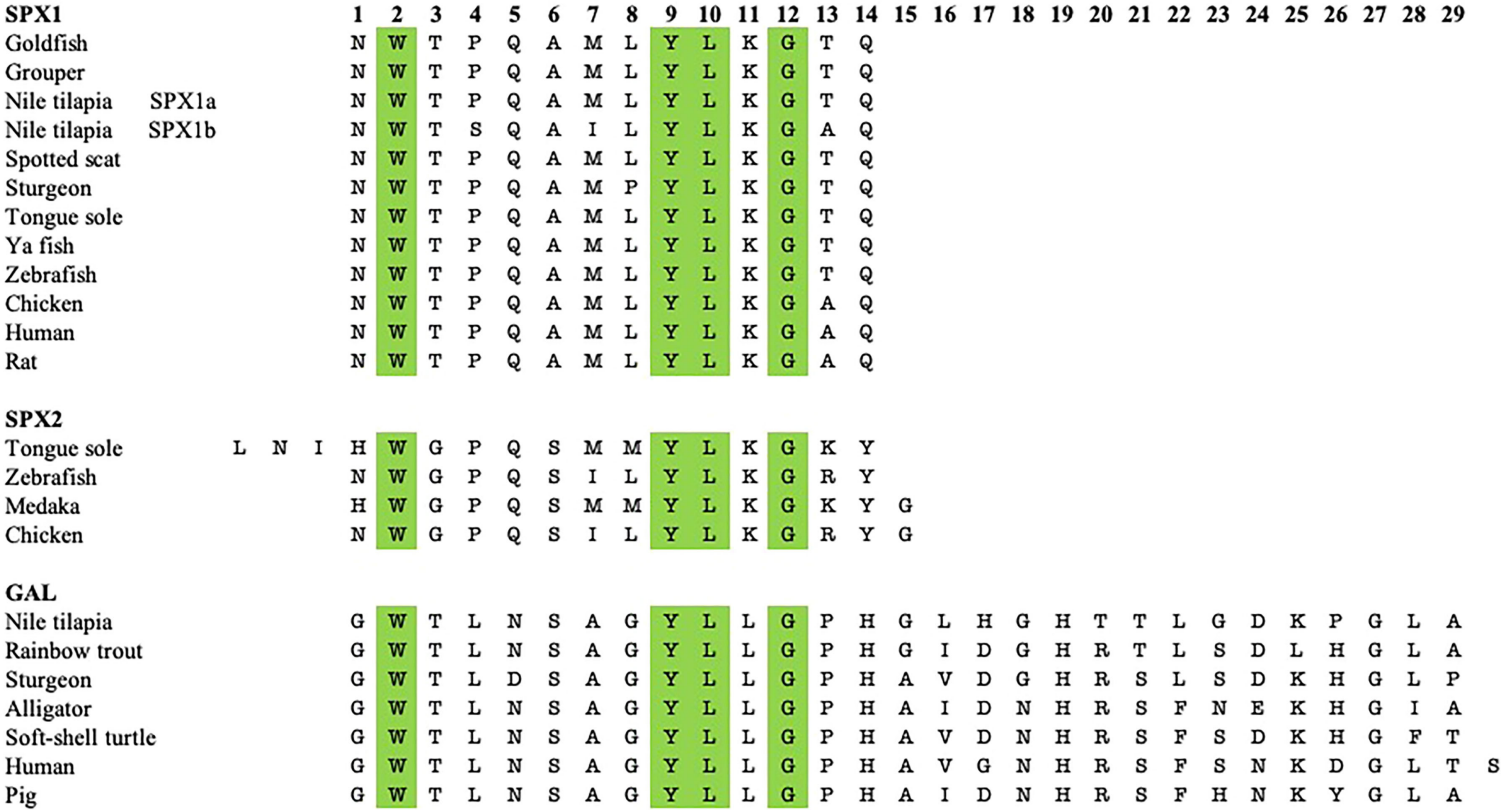
Sequence comparison between the mature peptide sequences of SPX1, SPX2, and GAL of various non-mammalian vertebrate species with some tetrapods. Both SPXs are 14 amino acids long, except for tongue sole SPX2, which is 17 amino acids long. GAL is mostly 29 amino acids long, Amino acids at positions 2, 9, 10, and 12 are conserved across the three neuropeptides.

SPX and GAL are evolutionarily conserved across vertebrate species. As shown in **Figure 1**, there is a high degree of similarity between the mature sequences of GAL and SPX in different vertebrate species. However, physiological significance of GAL and SPX, in particular, their clinical implications, remain unknown. On the other hand, studies using animal models including non-mammalian vertebrate species provide useful and beneficial information in biomedical research because of the high homology of these peptides in vertebrates. To date, functional characterization has revealed that GAL acts primarily as an orexigenic factor, as it is known to stimulate food intake (3, 4). In contrast, it has been implicated that SPX acts as a satiety factor (3). In addition to appetite regulation, GAL and SPX are involved in reproductive function such as in the regulation of gonadotropin and gonadotropin-releasing hormone (GnRH) release. SPX and GAL are also involved in stress response *via* the hypothalamic-pituitary-adrenal (HPA) axis (5). For example, GAL is co-localized in CRH neurons in some teleosts (6). Further, SPX and GAL expressions in the brain are sensitive to stress exposure, as seen in rats and Nile tilapia (7, 8). In addition, SPX and GAL have roles in modulating serotonin activity, indicating roles in mood disorders such as anxiety and depression (9, 10). The purpose of this review is to elaborate on the functional roles of SPX, GAL, and their receptors in metabolism and certain social behaviors in non-mammalian vertebrates, with a focus on fish species.

### 1.1 Galanin

**Figure 2:**
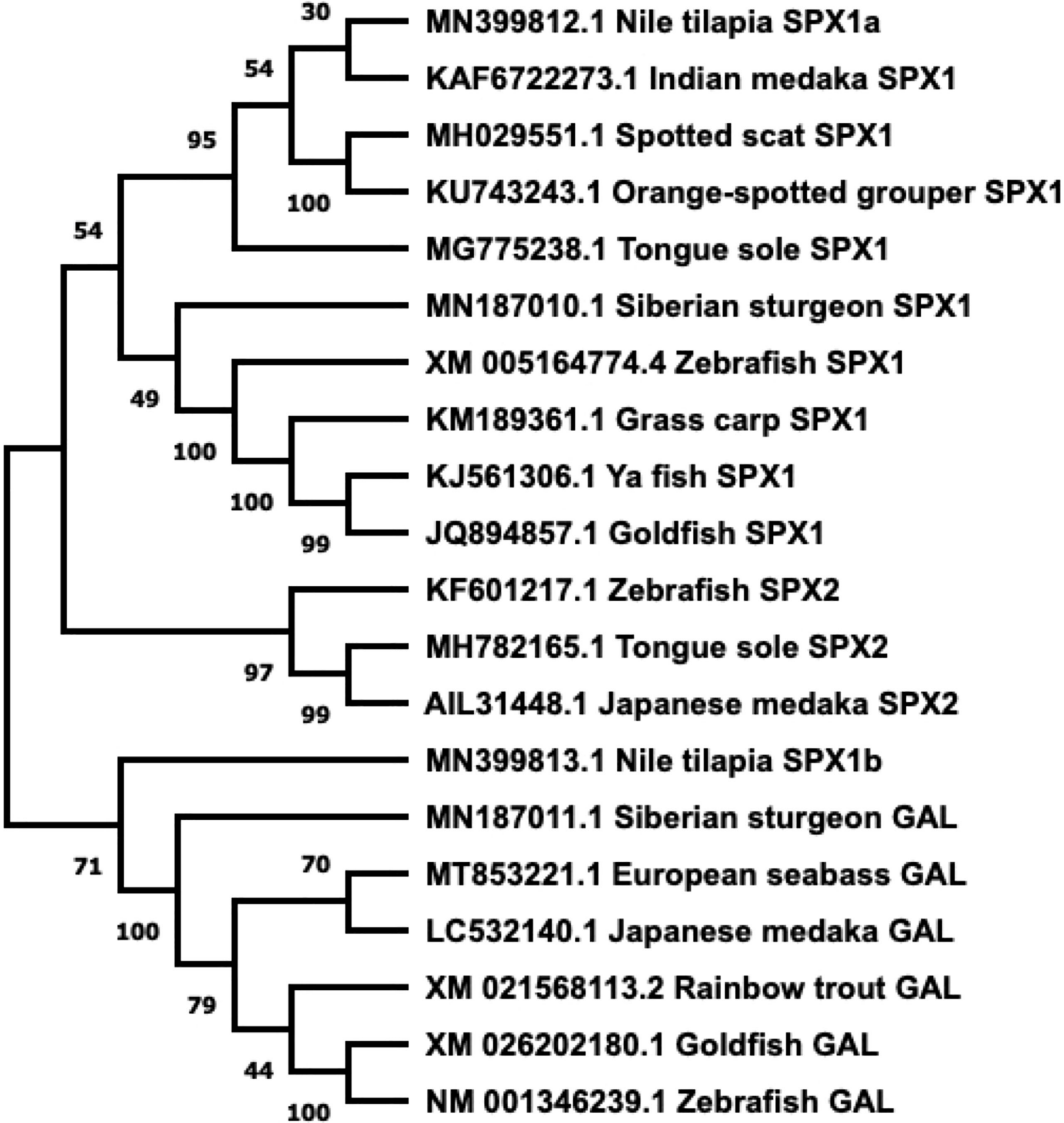
Phylogenetic tree of SPX and GAL present in fish. Sequences obtained from GenBank. Phylogenetic construction was done in MEGA X, using the Neighbor-Joining method. The percentage of the sequences clustered together is shown on the branches, out of 1000. SPX and GAL form two distinct branches, with SPX1 and SPX2 dividing further.

GAL was first isolated from the porcine intestine (11). Its name was derived from the glycine and alanine at its N- and C-terminals, respectively (11). In most species, including various fish species, GAL is 29 residues long, whereas it has 30 amino acids in humans (6, 12–14) (**Figure 1**). GAL binds to its receptors, aptly named GAL receptors (GALRs). Three forms of GALRs have been identified, namely GAL receptors 1, 2, and 3 (GALR1, GALR2, and GALR3, respectively) (15). Additionally, there are three other members of the GAL family: galanin message-associated protein (GMAP), galanin-like peptide (GALP), and alarin. GMAP is 59 amino acids long and comes from the preprogalanin precursor (16). GALP is a non-amidated 60 amino acid peptide with sequence similarity with GAL (17). It binds with high affinity with GALRs, with relatively lower affinity with GALR1, and was first isolated from porcine hypothalamus (17). Alarin was discovered recently as a splice variant of GALP. It was observed in the murine brain, thymus, and skin, while exhibiting vasoconstriction and anti-oedema activity. However, alarin’s activity is not mediated by any of the known GALRs. Thus, the cognate receptor for alarin still remains unknown (18). GAL-expressing cells are found in restricted brain regions, including the preoptic area and paraventricular hypothalamus, in various non-mammalian vertebrate species, as shown in **Table 1**. However, GAL fibers are widespread and extend all over the brain. A phylogenetic tree showcasing comparison between GAL and SPX gene sequences is shown in **Figure 2**.

**Table 1:** GAL-expressing cells were found in restricted brain regions of various non-mammalian species. However, GAL fibers were widespread and extended all over the brain.

	Rainbow trout	Green molly	Killifish	European seabass	Magadi tilapia	Flounder	Goldfish	Chicken	Japanese quail	Caspian turtle
	GAL	GAL	GAL	GAL	GAL	GAL	GAL	GAL	GAL	GAL
**Brain**										
Telencephalon	✓						✓			
Preoptic area	✓	✓	✓	✓	✓	✓	✓	✓	✓	✓
Mediobasal hypothalamus	✓							✓	✓	✓
Paraventricular hypothalamus		✓	✓	✓	✓	✓		✓	✓	
Periventricular hypothalamus									✓	✓
Ventromedial hypothalamus										✓
Tuberal hypothalamus		✓	✓	✓	✓	✓	✓	✓		✓
Amygdala										✓
Vagal lobe		✓	✓	✓	✓	✓				
Medulla								✓		
Lamina terminalis										✓
Hippocampus								✓		
Anterior commisure										✓
Septal area								✓	✓	
Solitary tract										✓
Pons									✓	

Localization information were obtained from: rainbow trout (12), teleost fish (Green molly, killifish, European seabass, Magadi tilapia, flounder) (6), goldfish (19), chicken (20), Caspian turtle (21), Japanese quail (22).

### 1.2 Galanin Receptors

**Figure 3:**
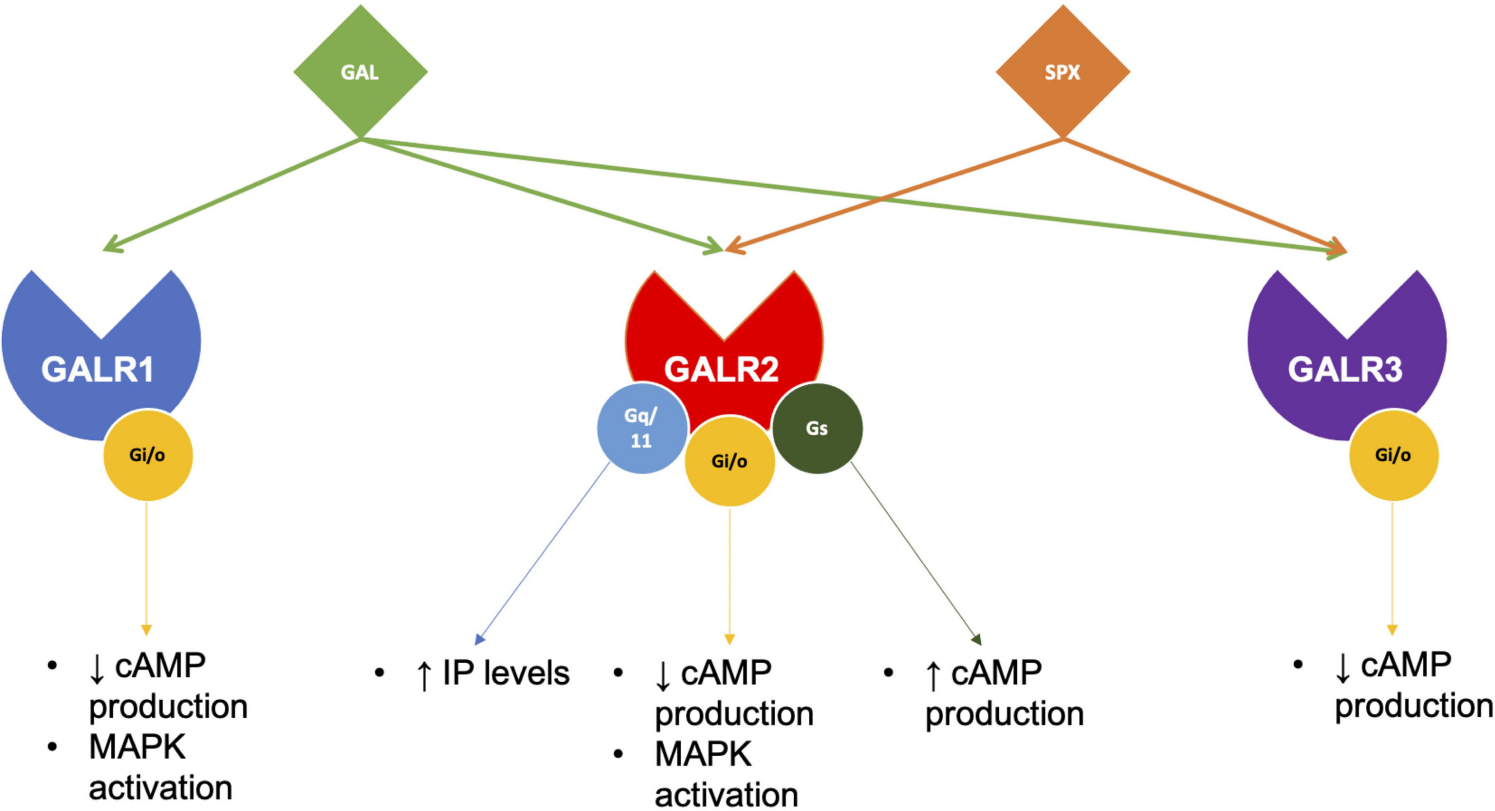
Galanin receptors pair with different G-proteins to execute SPX or GAL functions. In brief, GAL acts on all three of its receptors, while SPX only pairs with GALR2 and GALR3. Although both GALR1 and GALR3 can only pair with Gi/o proteins, GALR2 has been found to pair with several G-proteins, mediating various downstream effects.

**Figure 4:**
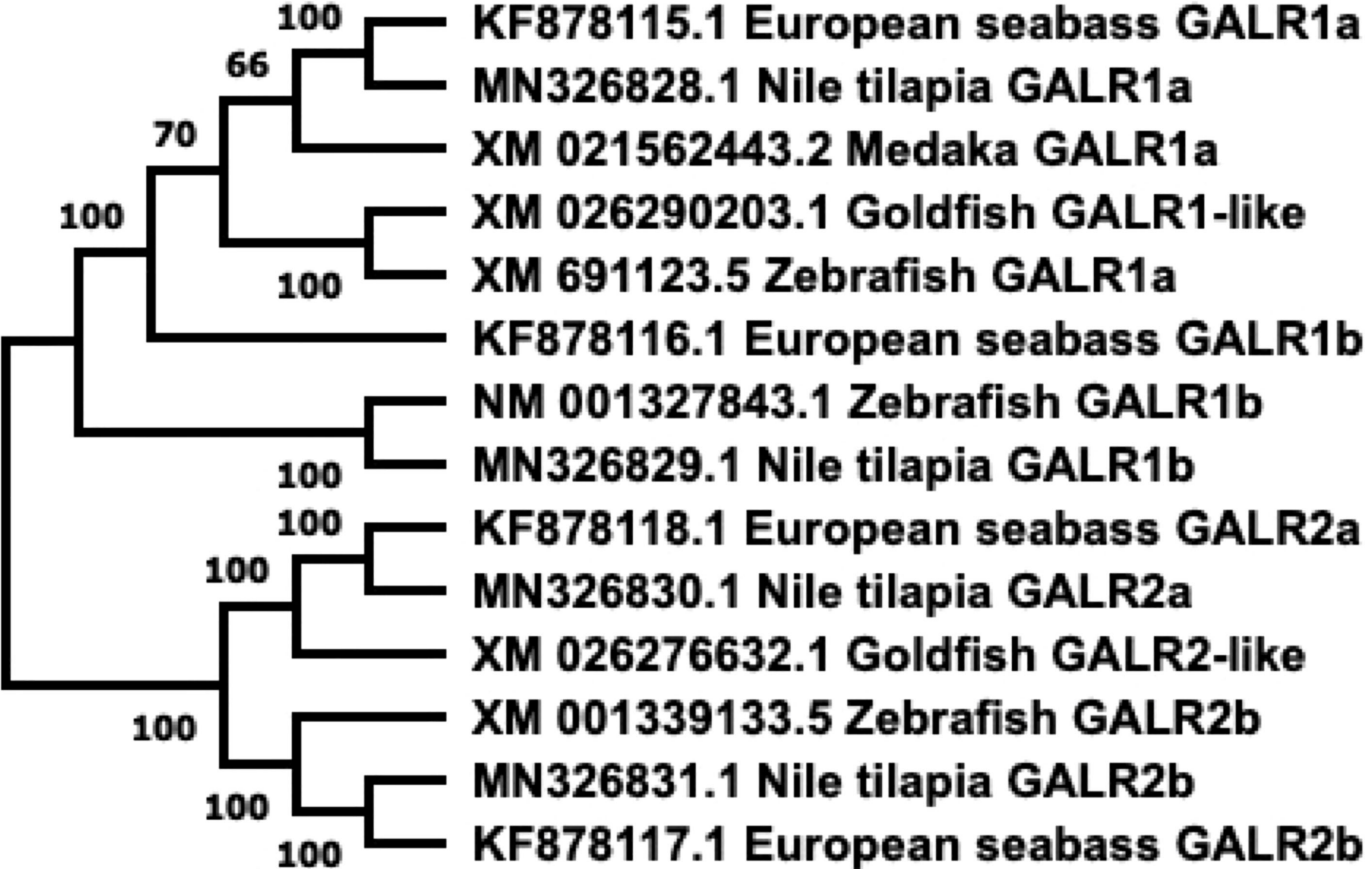
Phylogenetic tree of GALRs present in fish. Sequences obtained from GenBank. Phylogenetic construction was done in MEGA X, using the Neighbor-Joining method. The percentage of the sequences clustered together is shown on the branches, out of 1000 boots. GALR1 and GALR2 form distinct branches.

GALRs are responsible for mediating the functions of both SPX and GAL. GALRs are G-protein coupled receptors (GPCRs) that interact with G-proteins in cells to mediate their functions (**Figure 3**). In teleosts, there are two types of GALR1 types, namely GALR1a and GALR1b, as shown in **Figure 3** (23, 24). Similarly, GALR2 paralogs exist in non-mammalian vertebrates and are known as GALR2a and GALR2b (1, 23, 24). On the other hand, GALR3 is absent in non-mammalian vertebrates. GALR1 and GALR3 are known to interact with the G_i_-class of G-proteins and mediate inhibitory effects (25, 26). Interestingly, GALR2 is able to bind to different G-protein classes, which are G_s_, G_q/11_ and G_i/o_, and is able to mediate both stimulatory and inhibitory downstream effects (23). In mammals, GAL binds to all three GALRs, while SPX binds to GALR2 and GALR3. In fish, these receptors are localized in various regions in the brain and periphery (**Table 2**). Phylogenetic analysis of GALR gene sequence is detailed in **Figure 4**.

**Table 2:** Localization of galanin receptor expression in non-mammalian vertebrates.

	Nile tilapia				Zebrafish				European seabass				Chicken			
	GALR 1a	GALR 1b	GALR 2a	GALR 2b	GALR 1a	GALR 1b	GALR 2a	GALR 2b	GALR 1a	GALR 1b	GALR 2a	GALR 2b	GALR 1a	GALR 1b	GALR 2a	GAL R 2b
**Brain**					✓	✓	✓		✓	✓	✓	✓	✓	✓	✓	✓
Brainstem																
Cerebellum	✓	✓	✓	✓												
Hypothalamus	✓	✓	✓	✓				✓								
Medulla	✓	✓	✓	✓				✓								
Olfactory bulb	✓	✓	✓	✓				✓								
Optic tectum	✓	✓	✓	✓												
Preoptic area	✓	✓	✓	✓												
Spinal cord								✓								
Thalamus								✓								
**Peripheral**																
Pituitary	✓	✓	✓	✓									✓	✓	✓	✓
Gills	✓		✓			✓										
Heart	✓	✓	✓	✓											✓	✓
Intestine	✓	✓	✓		✓	✓							✓	✓	✓	✓
Kidney	✓	✓	✓	✓		✓							✓	✓	✓	✓
Liver	✓	✓	✓			✓									✓	
Lungs															✓	✓
Muscle	✓	✓	✓	✓											✓	
Ovary	✓	✓	✓	✓					✓	✓	✓	✓	✓	✓	✓	✓
Pancreas	✓	✓	✓										✓	✓	✓	✓
Retina	✓	✓	✓	✓		✓										
Spleen	✓	✓	✓			✓							✓	✓	✓	✓
Stomach	✓	✓	✓													
Testes	✓	✓	✓	✓					✓	✓	✓	✓			✓	✓

Information was found in research articles, references here: Nile tilapia (23), zebrafish (27, 28), European seabass (24), chicken (29, 30).

In a study looking at SPX binding to GALRs, SPX is found to exhibit high affinities to human, zebrafish, and *Xenopus* GALR2 and GALR3 (1), showcasing that SPX can activate GALR2 and GALR3 in mammalian and non-mammalian systems. Both SPXs, SPX1 and SPX2, can bind strongly to human GALR2/3. Although GALR2 has a higher binding affinity towards GAL as compared to SPX, GALR3 has a stronger preference for SPX binding than for GAL (1). Interestingly, SPXs have higher potency for zebrafish, GALR2b compared to GAL, while GALR2a interacts with both SPXs and GAL with similar affinities (1).

### 1.3 Spexin

SPX (also known as *C12ORF29*, according to its locus on human chromosome 12) was first discovered in the human proteome by bioinformatics (31–33). Since then, this peptide has been found in many other vertebrate species, including in teleost fish. A few paralogous forms of SPX have been identified. *Spx1a*, *spx1b*, and *spx2* are found in non-mammalian vertebrate genomes, whereas only *spx1* has been identified in mammals (1). It is likely that *spx* paralogues arose after two rounds of whole genome duplication in non-mammalian vertebrates (1). Additionally, cichlids express a paralogous *spx1b* gene, but not *spx2* (23). Both SPX genes encode for preprohormones that include a signalling peptide and the mature peptide flanked by dibasic cleavage sites. The mature SPX1 peptide sequence is 14 residues long and is conserved across species (34–39). Similarly, most known SPX2 sequences are also 14 amino acids long, except for the half-smooth tongue sole (*Cynoglossus semilaevis)* SPX2, which has 17 residues instead (1, 9, 37, 40). The mature peptide sequences of SPX1 and SPX2 are highly conserved across the species. Mature peptide and gene sequence comparison of GAL and SPX are depicted in **Figures 1**, **2**.

In teleosts, SPX neurons have been detected in various brain regions, as depicted in **Table 3**. In goldfish (*Cassasius auratus*), SPX-immunoreactive cells are located at the anterior hypothalamus, ventromedial thalamic nucleus, and medial longitudinal fasciculus (35). In the Nile tilapia (*Oreochromis niloticus*), SPX1a-expressing cells are found in the torus semicircularis (TS), while SPX1b-expressing cells are observed in the telencephalon (8). Comparatively, SPX expression in rats is particularly restricted to the mesopontine tegmentum (32). In a study focusing on the human magnocellular hypothalamus, SPX-expressing neurons were observed in the supraoptic and paraventricular nuclei (44).

**Table 3:** Distribution of SPX1 and SPX2 expression in the brain and periphery of fish species.

	Orange-spotted grouper	Zebrafish		Goldfish	Nile tilapia		Ya fish	Half smooth tongue sole		Spotted scat	Siberian sturgeon
	SPX1	SPX1	SPX2	SPX1	SPX1a	SPX1b	SPX1	SPX1	SPX2	SPX1	SPX1
**Brain**	✓	✓				✓	✓	✓		✓	
Brainstem				✓							
Cerebellum				✓					✓		
Habenula		✓									
Hypothalamus			✓	✓	✓				✓		✓
Medulla				✓					✓		
Optic tectum				✓	✓				✓		
Spinal cord									✓		
Telencephalon				✓		✓			✓		
Thalamus				✓					✓		
Torus semicircularis						✓					
**Peripheral**											
Pituitary	✓			✓		✓	✓	✓	✓	✓	
Gills	✓	✓		✓		✓	✓	✓	✓		
Heart		✓		✓		✓	✓	✓	✓	✓	
Intestine	✓	✓		✓	✓	✓		✓	✓	✓	✓
Kidney		✓		✓		✓	✓	✓	✓	✓	
Liver	✓	✓				✓	✓	✓	✓	✓	✓
Muscle	✓					✓	✓	✓	✓		
Ovary	✓	✓		✓	✓	✓	✓	✓	✓	✓	
Rectum											✓
Retina						✓					
Pancreas						✓	✓				
Skin							✓	✓			
Spleen	✓					✓	✓	✓	✓	✓	
Stomach	✓					✓			✓		✓
Testes					✓	✓	✓		✓	✓	

Localization was obtained for orange-spotted grouper (34), zebrafish (35, 40), goldfish (35), Nile tilapia (8, 23), Ya fish (41), half smooth tongue sole (37, 38), spotted scat (42), Siberian sturgeon (43).

In addition to *SPX* and *GAL*, *kisspeptin* (*KISS*) also descended from the same ancestral gene (1). Kim et al. found that the ancestral forms of these three genes are closely located on the same ancient chromosome (1). Kisspeptin is a neuropeptide encoded by the gene *KISS1* in humans and mammals, while orthologs exist in non-mammals, namely *kiss2* and *kiss3* (45). It can be speculated that *SPX*, *GAL*, and *KISS* genes arose from local duplications on the ancient chromosome before splitting into the many paralogous forms in modern vertebrate chromosomes after two rounds of whole-genome duplication. For example, *spx1* and *kiss2* are present on the same chromosome in zebrafish, anole lizard, and coelacanth, although in humans, *KISS2* is absent in the same corresponding chromosome. In the same species, *spx2* and *gal* are also localized in near vicinity but *SPX2* is absent in humans. Additionally, *galp* and *kiss3* are present on the same chromosome in the coelacanth and *Xenopus*, while *KISS3* is absent from the human genome (1).

The close relationship of SPX, GAL, and KISS means that they share similar functions. On that note, KISS has been implicated in metabolic functions, reproduction, mood, and behavior, similar to SPX and GAL. As SPX and GAL share receptors, this review will focus on these two neuropeptides and GALRs.

## 2 Physiological Functions of SPX and GAL

### 2.1 Metabolic Functions

#### 2.1.1 Spexin

SPX has been highly implicated in appetite regulation and food consumption in both mammals and non-mammalian vertebrates. In fish brains, different nutritional states affect *spx* gene expression. In the goldfish, *spx1* mRNA expression increases in the telencephalon, optic tectum, and the hypothalamus, as well as in the periphery after feeding (39, 46). The same effect is seen in the hypothalamus of Siberian sturgeon (*Acipenser baerii*), whereas in Ya-fish (*Schizothorax prenanti*), the increase is observed only in the forebrain (41, 43). On the other hand, *spx* expression is responsive to food deprivation, however, effects vary according to species, experimental design, and probably metabolic conditions. In the orange-spotted grouper (*Epinephelus coioides*), seven days of fasting caused an increase in hypothalamic *spx1* expression, with the levels recovering after refeeding (34). Similarly, *spx1* upregulation after food deprivation has also been observed in the spotted scat (*Scatophagus argus*) after two and seven days of fasting (42) and in the half-smooth tongue sole, although effects were only seen after 35 days of starvation and not after two or seven days (38). Additionally, in the tongue sole, *spx2* is upregulated after four weeks but not after two weeks of starvation (37). In contrast, other fish species such as goldfish, sturgeon, and Ya-fish display downregulation of *spx* gene expression after food restriction (39, 41, 43). In the brain of Nile tilapia, fasted adults have low expression levels of *spx1a, spx1b, and galr2a* but *galr2b* receptors remain unaffected (23). Thus, although feeding seems to clearly increase SPX expression, food deprivation instead has various outcomes in different fish species. This may be attributed to the different fasting duration between studies or to species-specific effects. The effects of different metabolic statuses on *spx* gene expression are detailed in **Table 4**.

**Table 4:** Summarised table of effects of metabolic status on central *spx* gene expression.

Status	Species	Duration	Effect on central *spx* gene expression		Ref
			*spx1*	*spx2*	
Unfed	Half-smooth tongue sole	35 days	Stimulated		(38)
		4 weeks		Stimulated	(37)
	Orange-spotted grouper	7 days	Stimulated		(34)
	Nile tilapia	26 days	Inhibited		(23)
	Ya-fish	1 week	Inhibited		(41)
	Spotted scat	2 days	Stimulated		(42)
		7 days		
Fed	Goldfish	3h	Inhibited		(39)
	Siberian sturgeon	3h	Stimulated		(43)
	Goldfish	1h	Stimulated		(39)
	Siberian sturgeon	1h	Inhibited		(43)
	Ya-fish	3h	Stimulated		(41)

Peripheral and central treatment of SPX in fish either reduces food consumption or causes change to appetite-regulating genes, or both, suggesting SPX as a satiety factor. Intraperitoneal (IP) or intracerebroventricular (ICV) injections of SPX suppresses food consumption in goldfish (39). SPX co-treatment with either neuropeptide Y (NPY) or orexin successfully block the appetite-stimulating effects of these two neuropeptides (39). ICV injections of SPX in the goldfish downregulates appetite-stimulating genes such as *npy*, *apelin* and agouti-related protein *(agrp)* in the brain (39). On the other hand, expressions of appetite-suppressing genes such as cholecystokinin *(cck*), proopiomelanocortin *(pomc)*, cocaine and amphetamine-regulated transcript *(cart)*, and melanin-concentrating hormone *(mch)* are upregulated following SPX treatment (39), suggesting SPX as an indirect appetite suppressant by acting on the aforementioned genes. Similarly, intracranial administration of SPX downregulates *agrp1* and *galr2a* expression, while *pomc1* and *galr2b* are significantly upregulated in zebrafish (47). Besides, IP treatment of SPX upregulates *pomc* mRNA expression in the hypothalamus, while at high doses, SPX downregulates *hcrt* expression in the orange-spotted grouper (34). IP treatment of SPX to the Siberian sturgeon results in an increase in anorexigenic factors nucleobindin 2 *(nucb2), cart*, urocortin 3 *(ucn3)*, and peptide YY *(pyy)*, while reducing *npy* expression in the hypothalamus (43). Effects of SPX treatment are summarized in **Table 5**.

**Table 5:** Details of SPX treatment in various animal models and mediums, with the effects in metabolism and reproduction.

Treatment detail	Species	Effects		Ref
		Metabolism	Reproduction	
Intraperitoneal	Goldfish	Reduce food intake	Suppresses LH release	(35, 39)
		Blocks appetite-stimulating effects of NPY and orexin	
		Downregulates appetite-stimulating gene expressions	
		Upregulates appetite-inhibiting gene expressions	
	Orange-spotted grouper	Upregulates pomc, downregulates hcrt		(34)
	Siberian sturgeon	Upregulates anorexigenic factors, inhibits npy expression		(43)
	Nile tilapia		Inhibits LH and FSH release	(23)
	Half-smooth tongue sole		Suppresses *fshb* gene expression	(38)
	Mice	Inhibits food intake		(48)
Subcutaneous	DIO rats	Inhibits food intake		(49)
Intracerebroventricular	Goldfish	Reduce food intake		(39)
	Mice	Inhibits food intake and lowers body weight		(50)
Intracranial	Zebrafish	Downregulates appetite-stimulating gene expressions		(47)
	*Spx1* gene KO zebrafish	Reduces food intake and agrp1 expression	
*In vitro*	Isolate human adipocytes	Stimulates lipolysis, inhibits lipogenesis and glucose uptake		(51)
	Cultured goldfish pituitary cells		Suppresses LH release	(35)

Furthermore, *spx1* gene knockout zebrafish exhibits an increase in *agrp1* expression in the brain and an increase in food intake, which is reversed by SPX1 intracranial treatment (47). It is clear from these studies in fish that SPX acts to increase satiety by increasing known satiety factors *via* downregulating appetite-stimulating genes. Interestingly, an IP injection of insulin into the goldfish resulted in an increase in *spx* expression in the liver and the brain (46) suggesting a possible link between food intake, insulin and SPX. It is possible that feeding-induced insulin surge triggers central *spx* gene expression to inhibit food intake *via* controlling both orexigenic and anorexigenic factors in the hypothalamus. Currently, there is limited information on the interaction between SPX and orexigenic and anorexigenic neurons in feeding behavior non-mammalian species.

In chicken, fasting results in rising blood SPX levels (52). The authors went on to investigate the expression of *spx* in tissues related to carbohydrate and lipid metabolism and discovered changes to *spx* and *galr* expressions. Thus, it seems that in chickens, both SPX and its receptors are affected by nutritional status, at least in the periphery (52). Treating rodents with SPX has an anorexigenic and weight loss effect (48–50, 53). IP treatment of SPX into mice inhibits food intake, even in mice fasted prior to treatment (48). ICV treatment into the third ventricle also inhibits food intake and lower body weight, without any changes to energy expenditure in mice (50). As SPX binds to GALR2 and GALR3, the former study identified hypothalamic GALR3 as the important receptor, resulting in suppression of *NPY* expression, whereas the latter study implicated GALR2 (48, 50). As further confirmation, wild type mice treated with SPX-based GALR2 agonist, known as SG2A, displayed a reduction in body weight and food consumption (54). A possible explanation to SPX’s body weight regulation is that SPX may prevent fatty acid uptake by adipocytes, as found in a study using mice adipocyte cells *in vitro* (49) or fatty acids could induce SPX, GALR2, and GALR3 expression as seen in mice hypothalamic cell lines (55). An increase in circulating fatty acids after a meal may act as a trigger for SPX-GALR2 and GALR3 signalling in the hypothalamus to reduce body weight and food consumption.

In diet-induced obese rats, subcutaneous SPX treatment is able to alter the food intake behavior, resulting in smaller portions and shorter mealtimes, possibly leading to weight loss (49). This suggests that SPX has potential as a therapy for weight loss. Interestingly, SPX is the most downregulated gene in fat from obese patients (49). In fact, circulating SPX levels are lower in obese patients compared to their normal-weight counterparts (49, 56, 57). This is also observed in type 2 diabetes patients (58). Further, *in vitro* SPX treatment stimulates lipolysis but inhibits lipogenesis and glucose uptake in isolated human adipocytes (51). Thus, it can be presumed that peripheral SPX is dysregulated in metabolic disorders, allowing for an increase in food consumption and body weight.

SPX is likely a satiety factor in vertebrates that acts centrally to mediate appetite-regulating genes and reduce food intake. Peripherally, SPX may act to regulate lipid metabolism. A dysregulation in SPX signaling can potentially affect food consumption behavior and adipose tissue regulation, leading to metabolic disorders. Using SPX as a treatment can likely counter these problems, although more studies are warranted to observe its benefits.

#### 2.1.2 Galanin

Contrary to SPX, GAL has been shown to stimulate feeding behavior. In the goldfish, preprogalanin (*gal*) mRNA are localized in the brain regions which are related to the regulation of food intake, namely in the area ventralis telencephali pars ventralis, nucleus preopticus periventricularis, nucleus lateralis tuberis, and the nucleus recessus lateralis, while GAL immunoreactivity has been detected in the gustatory nuclei of the vagal lobe (19, 59). Unfed goldfish have a significantly higher expression of *gal* mRNA in the telencephalon and hypothalamus (19). In young Japanese quails, 6 hours of fasting lowered *gal* expression in the hypothalamus compared to fed birds (60). In goldfish, ICV injections of GAL stimulate feeding behavior, which is blocked by a GAL receptor antagonist, galantide, and yohimbine, an α-adrenergic receptor antagonist (61). Similarly, in chicks, ICV GAL injections stimulate food intake, which is attenuated by co-treatment with yohimbine (62). Further, several studies have shown a GAL-induced increase in food consumption with GAL treatment into the PVN of rats (53, 63–66). This suggests that the α-adrenergic system may mediate orexigenic action of GAL *via* hypothalamic GAL neurons.

PVN GAL treatment also stimulates a preference for a high-fat diet while increasing carbohydrate metabolism and glucose uptake in muscles in rats (53, 66). In reverse, mice that were given a high-fat diet, plasma GAL as well as hypothalamic GAL and GALR1 expression increased (67). Rats treated with GALR1 selective agonist M617 show an increase in food intake (68). This suggests that GALR1 mediates GAL-induced feeding behavior in mammals. **Table 6** details the effects of GAL treatment.

**Table 6:** Summarised table of effects of various GAL treatments.

Treatment detail	Species	Effects				Ref
		Metabolism	Reproduction	Stress	Emotional control	
Intracerebroventricular	Goldfish	Stimulates feeding				(61)
	Chicken	Increase food intake				(62)
	Rat		Stimulates LH release			(69)
					Reduces 5-HT release	(10)
					Reduces 5-HT1A receptors in the dorsal raphe	(70)
					Increases immobility time	(71)
Into the PVN	Rat	Stimulates fat-diet preference				(53, 66)
*in vitro*	Rat pituitary cells		Stimulates LH release			(72)
	GnRH mice explants		Inhibits GnRH neuronal activity			(73)
Subcutaneous	Rat			Increases ACTH, aldosterone, corticosterone levels		(74)

Studies in humans have shown the role of GAL in metabolic disorders. Obese patients and patients experiencing gestational diabetes mellitus have significantly higher plasma levels of circulating GAL (75–77). As orexigenic peptide NPY immunoreactive fibers innervate GAL immunoreactive cells in the hypothalamus of the human brain (4), it is possible that NPY can affect plasma GAL levels in metabolic conditions, as the NPY system is dysregulated in obese conditions (78).

### 2.2 Social Behaviors

#### 2.2.1 Spexin

##### 2.2.1.1 Reproduction

SPX neurons and SPX receptors, GALR2a and GALR2b have been observed in the preoptic area of several fish species, indicating a possible role in the reproductive axis (8, 23, 35). SPX was first implicated in reproductive function because it suppressed LH release from cultured goldfish pituitary cells in an *in vitro* study, as well as after IP injections in an *in vivo* study performed on goldfish (35). This was also observed in the Nile tilapia, where IP injections of SPX inhibited LH and FSH release (23). It is likely that SPX exerts inhibitory effect on gonadotropin release. At the transcriptional level, there are species-specific effects of peripheral SPX treatment on central gene expression. IP injections of SPX does not affect *fshb* and *lhb* mRNA expression in the pituitary of the orange-spotted grouper (34). In the half-smooth tongue sole, high dose of SPX treatment suppressed *fshß* expression but not *lhß, gnih*, *gnrh2/3* and *orexin* in the hypothalamus (38). Thus, peripheral SPX might have a role in affecting LH or FSH release in some teleost species. It will be beneficial to uncover the role of central SPX in reproduction. Additionally, as SPX receptors are localized to the preoptic area, there is a possibility that central SPX may act on reproductive-related neurons in this region. Furthermore, brain *spx* expression is low during the breeding season but is high during development and puberty in the grouper and goldfish (34, 35). This suggest SPX is suppressed during the breeding season to facilitate reproductive function mediated by gonadal hormones, as estradiol treatment downregulates *spx* expression (35, 42). On the other hand, high expression of *spx* during development and puberty is likely to prevent premature LH/FSH release. Despite the evidence, *spx1* knockout transgenic zebrafish did not show changes to reproductive capability, puberty onset, or gamete maturation (79). It is likely that the loss of SPX signalling in knockout zebrafish is compensated by other neuropeptides involved in the control of reproduction in fish (79). More evidence from GALR2 or GALR3 knockout studies may lead to clearer understanding of the role of SPX in the reproductive system. Importantly, fatty acids are able to induce SPX, GALR2, and GALR3 expression in a mice hypothalamic-GnRH cell line (80), which could be similar to an obese condition. If SPX does negatively regulate the reproductive axis in mammals, then the increase in SPX expression could partially explain the underlying obesity-induced reproductive dysfunction (81). In women with the reproductive disorder, polycystic ovarian syndrome (PCOS), circulating SPX levels are low compared to women with normal menstrual cycles (82). It is, therefore, possible that at least peripheral SPX is involved in the pathophysiology of PCOS, which often have high levels of LH secretion. Although some studies have suggested the effect of SPX on LH release in the pituitary, the function of SPX neurons, SPX-GAL2/3 signaling on reproduction-related neurons in the brain remains unclear.

##### 2.2.1.2 Stress Response

Stress-activated HPA axis exerts inhibitory action on the reproductive system. SPX has been implicated in stress response. Chronic stress exposure in the Nile tilapia show high cortisol levels, low reproductive activities, and upregulation of *spx1a/1b* expression in several parts of the brain, namely, in the midbrain, hypothalamus, and optic tectum (8). In mice, chronic unpredictable stress exposure and CRH treatment reduces SPX gene expression in the hippocampus, hypothalamus, and pituitary (83). Therefore, it is likely that different types of stress might have varying effects on site-specific SPX1 expression in the brain.

##### 2.2.1.3 Emotional Control

Stress-activated HPA axis also exerts emotional control in the brain. SPX1 neurons in the dorsal habenula project to GALR2a/2b expressing cells in the interpeduncular nucleus (IPN) of the zebrafish (27, 40). Transgenic zebrafish that overexpress *spx1* in the dorsal habenula show reduced anxiety but increased serotonergic-related genes in the raphe compared to wild-type (9). The IPN project fibers to the raphe. Therefore, it is likely that SPX1 mediates anxiety and induces anxiolytic effects by modulating the serotonergic system *via* GALR2a/2b in the IPN of zebrafish (9).

SPX-based GALR2 agonist relieves anxiety-like and depressive-like symptoms in mice (54, 84) as in zebrafish (9). Chronic stress in mice is associated with an increase in *CRH*, a decrease in *spx* expression in the hippocampus and a display of anxiety-like behavior (83). These studies suggest that SPX is involved in mood and negative emotions.

#### 2.2.2 Galanin

##### 2.2.2.1 Reproduction

GAL neurons have been identified in the preoptic area of several fish species, including Xiphophorus, rainbow trout, brown ghost fish, green molly, sailfin molly, European sea bass, flounder, killifish, and tilapia (6, 85–87), with fiber projections to the entire brain and gonadotrophs in the pituitary (6, 88).

*GAL* gene expressions in the brain and gonads are differently regulated by sex steroids production in male and females in sea bass (89). The sailfin molly displays sexual dimorphic localization of GAL fibers on the brain. Males have a higher number of GAL immunoreactive fibers in the optic tectum, torus semicircularis, brainstem tegmentum, spinal cord, while in females fibers are limited to the ventromedial tegmentum and trigeminal area (87). Similarly, sexual dimorphism in GAL expression is observed in the goldfish, red salmon, and brown trout (90–92). Specifically, gender-specific differences in GAL immunoreactivity at the preoptic area has been observed in the molly, goldfish, and red salmon (87, 90, 91). GAL neurons in the preoptic area are stimulated during mating behavior in teleost, indicating the involvement of the GAL circuitry in reproductive-related social behavior (93).

Sexual dimorphism of GAL expression is also observed in the rat but not in the ovine brain (94, 95). In sheep, all GnRH cells co-express GAL, suggesting GAL’s significant role in the reproductive axis (95). Additionally, GnRH neurons express all three GALRs with evidence of GAL synapsing directly onto GnRH cell bodies in rodents (73, 96, 97). ICV administration of GAL into rats primed with estradiol benzoate and progesterone results in an increase in plasma LH levels, while pre-treatment with galantide, a non-specific GALR antagonist, blocked the increase (69). GAL treatment directly on pituitary cultures from rats upregulates basal LH secretion in a dose-dependent manner, while co-treatment with GnRH potentiates GnRH-induced LH secretion *in vitro* (72). Thus, it seems that GAL has a stimulatory effect on LH release, likely by directly influencing the pituitary. In addition, GAL may be able to modulate GnRH activity. A study in mice identified GAL as a regulator of GnRH release, by modulating kisspeptin-induced GnRH stimulation. GAL acts to reduce kisspeptin-induced GnRH neuronal activation *in vitro* (73). Therefore, GAL has a possible inhibitory effect on GnRH release when directly acting on GnRH neurons *via* GALR1, probably acting as a balancing factor for GnRH neuronal activation. A cluster of GAL-expressing cells in the medial preoptic area (mPOA) is activated during parental behavior, while a separate cell population in the same area is active during mating. Ablation of mPOA GAL cells results in loss of parental behavior in both male and female rats. These results show the importance of GAL of mPOA in social behavior related to reproduction and offspring care (98).

Intravenous infusion of GAL to women does not change LH and FSH levels but enhance growth hormone release (99). In women with PCOS, there is an increase in GAL immunoreactive fibers that innervate polycystic ovaries; however, there is no significant difference in plasma GAL levels (100, 101). Thus, in humans, the role of GAL in reproductive disorders remains largely unknown.

##### 2.2.2.2 Stress Response

In some teleost fish, namely sailfin molly and killifish, GAL immunoreactivity is co-localized in CRH-like-immunoreactive cell bodies, implicating the involvement of GAL with the hypothalamic-pituitary-adrenal axis (6). However, social stress does not affect the expression of GAL mRNA in the brains of zebrafish (102).

Rats exposed to immobilization stress show upregulated GAL protein levels and intense GAL immunoreactivity in the hypothalamus and increased GAL mRNA in the adrenal gland (7). Stress because of exposure to predator scent reduces GAL mRNA levels in the frontal cortex and hippocampus and induces anxiety-like behavior. Further, the anxiety-like behavioral response is reduced when galnon, a GAL receptor agonist is given immediately after exposure to predator scent (103). In rats, immobilization stress and formalin injections increase GAL mRNA levels in the hypothalamus (104), and chronic social stress upregulates the expression of p*Gal* mRNA in the locus coeruleus (105). On the other hand, subcutaneous GAL injections increase adrenocorticotropic hormone (ACTH), aldosterone, and corticosterone levels (74). Central injections of GAL stimulate CRH release from the rat hypothalamus in a dose-dependent manner (106). Allowing mice to freely exercise results in an elevation of locus coeruleus-derived GAL and resistance to anxiogenic conditions, suggesting that GAL could be mediating stress resilience (107). Overall, it seems that in stressful conditions, GAL upregulation can stimulate the HPA axis to release stress hormones. This suggests that GAL can trigger the body’s stress response.

##### 2.2.2.3 Emotional Control

In rodents, there is a clear involvement of GAL in anxiety and depression. Firstly, GAL is co-expressed in serotonergic neurons in the dorsal raphe of rats (108, 109). GAL exerts an inhibitory influence on the noradrenergic and serotonergic systems (10, 70, 110). GAL treatment reduces serotonin release into the ventral hippocampus, and receptor antagonist treatment blocks this effect (10). Similarly, pre-treatment with a GALR2 antagonist blocked the anxiogenic effects of GAL administration into the dorsal hippocampal area (111). It is likely that GAL affects serotonin synthesis, as GAL significantly reduces the mRNA expression of tryptophan hydroxylase in the dorsal raphe nucleus (10). GALR1 mediates pro-depressive effects of GAL after stress exposure by affecting the expression of the serotonin receptor, 5HT_1A_R (112). ICV GAL treatment mediates pro-depressive effects, which are overcome by its antagonist treatment (71, 112). ICV treatment of GAL, GALR1 agonist or GALR2 antagonist increases the immobility time, and GALR2 agonist decreases the immobility time of rats in the forced swim test. This indicates differences in the activity of GALR1 and GALR2, in which GALR1 may be pro-depressive while GALR2 mediates GAL’s anti-depressant activity (112). In addition to that, a GALR2 agonist mediated the anti-depressive effects of GAL when treated into the dorsal raphe nucleus, which was blocked by its antagonist (113). However, as GALR2 can induce different cellular signalling pathways, therefore, it is not impossible for GALR2 to mediate pro-depressive effects. It is also likely that GALR3 has pro-depressive effects, since an antagonist treatment results in anxiolytic and anti-depressive effects, by reversing GAL’s inhibition of serotonin release and transmission in the hippocampus (114). These findings in rodents indicate GAL’s possible role in developing and mediating depression and anxiety.

Interestingly, in humans, certain gene variants of GAL and their receptors confer vulnerability towards psychosocial stress and increase the risk of developing anxiety and depression (115). The activity of GAL is more prominent in a highly stressed population; GALR1 SNPs are linked to negative childhood events while GALR2 and GALR3 mediate effects of recent negative experiences. Thus, it is likely that GAL has a crucial role in developing depression, particularly if a person experiences childhood trauma or recently experienced negative events (115).

### 2.3 Role of Galanin Receptors

As discussed above, SPX and GAL show opposing actions and functions in the metabolic, reproductive system and emotional control. The likely reason to this is the existence of different receptors. It is interesting that GAL and SPX share receptors. However, it is difficult to distinguish if GALR2 or GALR3-linked effects are due to GAL or SPX binding. Different ligand-receptor interactions may lead to different downstream signaling. For example, GALR1 activation results in pro-depressive effects while GALR2 stimulation is linked with anti-depressant activity (71). GAL is known to be pro-depressive; thus, it must have acted on GALR1 receptors. In contrast, GALR2 anti-depressant effects can possibly be due to SPX activation instead of GAL. GALRs affinities towards the two ligands may dictate which neuropeptide ends up binding and inducing its action. For example, GALR3 has a higher affinity for SPX than GAL (1), thus, GALR3-related actions are likely the result of SPX binding. SPX and GAL interaction with other circulating factors, such as neurotransmitters or neuropeptides, may also influence downstream effects. On top of that, upstream regulation or external factors of SPX and GAL, such as fatty acids or steroid, certain physiological conditions and states, or other neuropeptides, can likely determine which neuropeptide will exert its action on which receptor. Accordingly, estradiol, as an upstream regulator, can suppress *SPX* expression in the spotted scat hypothalamus (42) while GAL expression is induced in GnRH neurons of rats (116). The complexity increases with GALR2’s ability to bind to different G proteins. A possible speculation is that GALR2-G-protein interaction is dependent on cell type although this warrants further investigation.

In relation to appetite regulation, GALR1 activation lead to increased food intake as per GAL’s role (68). Restricted feeding increased hypothalamic GALR2 in mice while GALR2 activation reduced food intake (54, 67). In addition, there is the likelihood that SPX may act on hypothalamic GALR3 to suppress appetite-stimulating gene expression, meaning that SPX can act *via* both GALR2 and GALR3 as a satiety factor (48). In zebrafish, GALR2b upregulation with consequent GALR2a reduction in the brain is linked with satiety (47). This suggests GALR2b as SPX’s receptor in appetite regulation in non-mammalian vertebrates.

Single cell analysis of GnRH neurons identified GALR1 expression, but not GALR2 or GALR3 (73). In another study, GALR1 and GALR2 were detected in mice GnRH cells (117). However, it was found that GALR2 and GALR3 had low basal levels and were stimulated by fatty acids *in vitro* (80). It is possible that physiological levels of GALR2 is low in GnRH neurons and can be stimulated during abnormal conditions, such as high circulating fatty acid levels. At this point, it is unknown if GAL or SPX can act on GALR2 to mediate dysfunction on GnRH cells. GALRs are also expressed in the pituitary, and both SPX and GAL act to suppress or stimulate LH/FSH release *in vitro*, respectively (35, 69).

GALR2 is the likely receptor for SPX-induced anxiolytic functions. This seems to be the case in zebrafish and mice (9, 54, 83). In fact, a SPX-based GALR2 agonist was developed and can induce anxiolytic effects as well as regulate appetite, even when given intranasally (54, 118). Most studies implicate GALR2 as an anti-depressive, while in contrast, GAL likely acts on GALR1 and GALR3 to induce changes to the serotonergic system, resulting in pro-depressive effects (70, 71).

## 3 Summary

In summary, SPX and GAL are two neuropeptides that have been receiving considerable interest in metabolic and reproductive medical research. GALRs mediate the actions of both SPX and GAL. While GAL can bind to all three GALRs, SPX can only act on GALR2 and GALR3. A significant amount of evidence describes the involvement of SPX and GAL in metabolic functions and social behaviours, including reproduction, stress responses and emotional control in non-mammalian and mammalian vertebrates. SPX acts as a satiety factor and is affected by nutritional conditions, while GAL is an orexigenic factor. SPX inhibits gonadotropin release, while GAL modulates gonadotropin release by acting on the pituitary or on GnRH neurons. SPX and GAL are involved in the body’s stress response. Further, SPX and GAL are also involved in depression and anxiety, where studies in fish have shown a possible anxiolytic function of SPX. Although lacking in fish studies, GAL’s pro-depressive and anxiety functions in rodents is clear; however, its effects depend on the receptor it interacts with. GAL and SPX are important neuropeptides for feeding, energy metabolism, reproduction, and emotional control *via* the correlation with other neurons and the three GALRs in the brain. GAL and SPX likely contribute to balancing the functions of the complex neuroendocrine system. In the future, more understanding of the physiological effects of GAL and SPX with three GALRs could lead to the development of new therapeutic strategies such as neuroendocrine-based obesity elimination and reproductive abnormalities using GAL and SPX.

## Author Contributions

IMZ wrote the initial draft of the manuscript and drew the figures. TS, SO, and NAD edited the manuscript. ISP conceptualized and edited the manuscript. All authors contributed to the article and approved the submitted version.

## Funding

This work was supported by the JCSMHS SEED GRANT SED-000082 (2020) (TS*, SO, NAD, IMZ, ISP) from School of Medicine and Health Sciences, Monash University Malaysia. IMZ is the recipient of Monash University Malaysia Graduate Research Merit Scholarship.

## Conflict of Interest

The authors declare that the research was conducted in the absence of any commercial or financial relationships that could be construed as a potential conflict of interest.

## Publisher’s Note

All claims expressed in this article are solely those of the authors and do not necessarily represent those of their affiliated organizations, or those of the publisher, the editors and the reviewers. Any product that may be evaluated in this article, or claim that may be made by its manufacturer, is not guaranteed or endorsed by the publisher.
